# Two genetic variants in *NEXN* and *ABCC6* genes found in a patient with right coronary artery to right ventricle fistula combined with giant coronary aneurysm and patent ductus arteriosus

**DOI:** 10.3389/fcvm.2022.1048795

**Published:** 2022-11-17

**Authors:** Yongxuan Peng, Jiajun Ye, Yuejuan Xu, Jihong Huang, Yurong Wu, Wei Liu, Kai Bai, Sun Chen, Yanan Lu

**Affiliations:** ^1^Department of Pediatric Heart Center and Pediatric Cardio-Thoracic Surgery, Xinhua Hospital, Affiliated to Shanghai Jiao Tong University School of Medicine, Shanghai, China; ^2^Department of Pediatric Heart Center and Pediatric Cardiology, Xinhua Hospital, Affiliated to Shanghai Jiao Tong University School of Medicine, Shanghai, China

**Keywords:** coronary artery fistula, coronary artery aneurysm, coronary artery development, *NEXN* gene, *ABCC6* gene

## Abstract

**Objective:**

Coronary artery fistula, defined as communication between a coronary artery and a great vessel or a cardiac chamber, is a relatively rare anomaly with an estimated incidence of 0.002% in the general population. It could be combined with a giant coronary artery aneurysm, with an incidence of 5.9% of the total incidence rate of CAF in the general population. The pathogenesis of these two combined anomalies is not clear, and we aimed to detect whether genetic abnormalities underlie the pathogenesis of these rarely combined anomalies.

**Materials and methods:**

A 6-year-old patient with a diagnosis of the right coronary artery to right ventricle fistula combined with a giant right coronary artery aneurysm and patent ductus arteriosus underwent a surgical repair at our center. The diagnosis was confirmed by echocardiography, CT, and surgery. DNA was extracted from the peripheral venous blood samples of the patient and his mother after informed consent was obtained. Hematoxylin and Eosin (HE) and Alizarin red staining were performed on the excised coronary artery aneurysm. Exome sequencing and *in silico* analyses were performed to detect detrimental genetic variants.

**Results:**

No obvious abnormalities were found in the excised coronary artery aneurysm. A heterozygous truncated variant (NM_144573: c.G298T; p.G100X) in the *NEXN* gene and a missense variant (NM_001171: c.G1312A; p.V438M) in the *ABCC6* gene were carried by the patient but not by his mother.

**Conclusion:**

The NEXN-truncated variant, NEXN-G100X, is associated with the development of coronary arteries and congenital coronary artery anomalies.

## Introduction

Coronary arteries (CAs) are blood vessels that nourish beating heart muscle cells, cardiomyocytes. Proper coronary circulation is important for myocardial homeostasis and cardiac function. Disruption of coronary development during embryogenesis leads to congenital coronary defects, including anomalies of CA connection, anomalies of intrinsic CA anatomy, and anomalies of the myocardial/CA interaction. Coronary artery fistulas (CAFs) are a relatively rare congenital coronary anomaly with an estimated incidence of 0.002% in the general population and 0.1–0.2% among patients undergoing coronary angiography (1). CAFs are defined as communication between a coronary artery and a great vessel or a cardiac chamber (2, 3). Most CAFs are congenital and account for 0.2–0.4% of congenital cardiac abnormalities (4). CAFs most commonly affect the right side of the heart (1). The majority of CAFs arise from the right coronary artery (50–60% of cases), and less from the left anterior descending artery (25–42%) and the left circumflex artery (18.3%) (1). The drainage site of CAFs is often in the right heart, usually, the right ventricle (14–40% of cases) and pulmonary artery (15–43%), followed by the right atrium (19–26%), left ventricle (2–19%), coronary sinus (7%), left atrium (5–6%), and superior vena cava (1%) (1). Normally, high pressure inside the ascending aorta drives blood flow into both coronary arteries, from the main stem to the distal branches, and finally to the capillary network inside the myocardium. When CAFs exist, the fistula works as an abnormal shunting channel, leading the coronary artery blood flow into the cavity connecting with the fistula rather than the myocardial capillary network. Therefore, CAFs can lead to myocardial hypoperfusion and volume overload of the affected ventricle, eventually leading to cardiac dysfunction. Surgical indications for CAFs are controversial to some degree because there is no rigid standard for surgical repair. However, there is some consensus; when the fistula is small (less than 1 or 2 mm) without heart murmur or dilation of the heart, follow-up observation is recommended, and when the fistula is large (>3 or 4 mm) with a significant heart murmur and dilation of the heart, surgical repair is necessary.

A coronary artery aneurysm (CAA) is defined as a coronary dilatation that exceeds 1.5 times the diameter of the normal adjacent lumen and is considered a giant CAA if it exceeds 20 mm in diameter (5). The pathogenesis of CAA has not been well elucidated. Causative factors include atherosclerosis, Takayasu arteritis, congenital disorders, Kawasaki disease (KD), and percutaneous coronary intervention (6). The reported incidence of coronary aneurysms ranges from 0.3 to 5% in patients undergoing coronary angiography (7). Giant CAA are rare, with an estimated incidence of 0.02% (8). CAFs combined with giant CAA are rare, with an incidence of 5.9% among patients with CAFs (9). The prognosis for CAFs with giant CAA is poor. The formation of congenital CAF and CAA remains poorly understood (9).

Our current understanding of coronary blood vessel development is mainly based on the results of studies in mouse models, which provide a dynamic approach to coronary embryonic development and crucial information on the origin, fate, and patterning of CA (10). Several genes such as *VEGF-A/C* and *Tbx1* have been reported to be important for proper coronary artery development (10). Congenital coronary anomalies are present in genetic syndromes, such as Marfan syndrome (11) and Loeys-Dietz syndrome (12). However, the causes of congenital coronary anomalies remain unknown. Here, we report a rare association between congenital CAF and CAA and isolated patent ductus arteriosus (PDA) in a 6-year-old boy who underwent a surgical repair. Genetic evaluation and testing revealed that the patient harbored a novel truncated variant G100X in NEXN, which is an F-actin binding protein at cell–matrix junctions and is implicated in atrial septal defect (ASD) and cardiomyopathies (13).

## Materials and methods

### Ethics statement

The study protocols were approved by the Xinhua Hospital Ethics Committee (XHEC-C-2019-083). Signed consent forms were obtained from all participants. This study conformed to the principles outlined in the Declaration of Helsinki.

### Case and phenotype description

**FIGURE 1:**
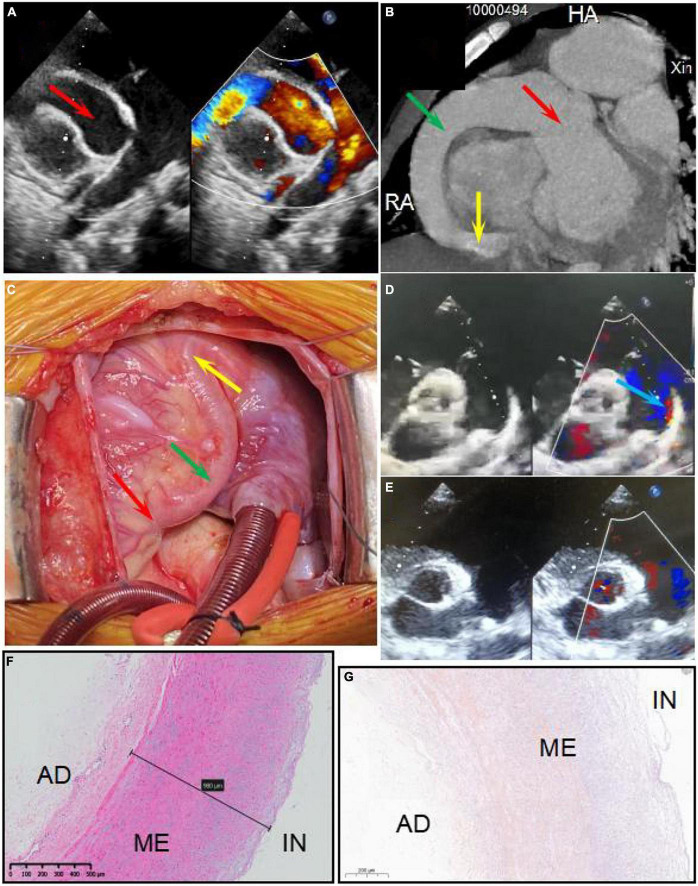
Panels **(A,B)** show the right coronary artery fistula (RCAF) by echocardiogram and CT, respectively. Panel **(C)** shows the intraoperative photograph of the RCAF. The red arrow shows dilated ostium of RCA. The green arrow shows dilated the main stem of RCA. The yellow arrow shows the fistula. The blue arrow shows the patent ductus arteriosus (PDA). Panels **(D,E)** show the echocardiogram of PDA before surgery and after surgery, respectively. Panel **(F)** shows the HE staining of the excised coronary artery aneurysm. The structure of the coronary artery is almost normal. Panel **(G)** shows the negative calcium slats accumulation by Alizarin red staining of the excised coronary artery aneurysm. IN, intima; ME, media; AD, adventitia.

A 6-year-old boy was admitted to Xinhua Hospital, affiliated with Shanghai Jiao Tong University School of Medicine, with complaints of right coronary artery fistula (RCAF), which was diagnosed when he was 1 year old at a local hospital due to a heart murmur. His face looked normal, no other extracardiac abnormalities were detected, and there was no pedigree or family history of any significant cardiac ailments in his parents’ families. He was asymptomatic except for being scrawny and was receiving non-scheduled follow-ups without therapy before coming to our center, based on the local doctor’s advice. Physical examination revealed normal vital signs with a continuous murmur. Transthoracic echocardiography revealed a large fistula extending from the right coronary artery to the right ventricle (Figure 1A). Cardiac electrocardiogram-gated computed tomography coronary angiography confirmed that the aneurysmal right coronary artery arose from the right aortic sinus and terminated in the right ventricle (Figure 1B). Intraoperatively, an aneurysmal right coronary artery was observed (Figure 1C). The right coronary artery passed through the right posterior atrioventricular groove and inverted into the right ventricle below the tricuspid valvular annulus with a 7 mm fistula. The entire right coronary artery was dilated to 21 mm, extending from the ostium of the right coronary sinus to the distal end close to the fistula. In addition, the parasternal short-axis view of echocardiography showed patent ductus arteriosus with a shunt bundle 1.0 mm wide with a flow rate of 2 m/s (Figure 1D).

An interdisciplinary discussion on the therapeutic strategy for this patient was conducted with surgeons, cardiologists, and intensivists at our heart center. An operation was considered appropriate for the following reasons: First, the fistula was very large with many left-to-right shunts, so there was little chance that the fistula would close or reduce spontaneously. Second, the patient was scrawny for a long time, indicating that the CAF affected his growth and development. Last, the echocardiography found there was a giant CAA, indicating a risk of coronary artery thrombosis. The patient underwent a median thoracotomy. During the surgery, the entire section of the right coronary artery was found to be significantly dilated, with a diameter of 21 mm (Figure 1D). After incising the anterior wall of the right coronary artery, the fistula was found to be approximately 8 mm and entered the right ventricle near the right posterior atrioventricular groove. The fistula was repaired using one pericardium patch, while the whole segment of the right coronary artery, including the ostium, was volume-reduced. The patent ductus artery was approximately 3 mm in size and ligated. Parasternal echocardiography showed no shunt after surgery (Figure 1E). Ventilator weaning was delayed due to severe pneumonia and heart dysfunction after surgery. The ventilator was withdrawn on the fourth-day post-operation, and the patient was transferred from the ICU to the general ward 2 days later. Echocardiography showed that while the left ventricular diastolic diameter (LVDD) was significantly reduced due to the elimination of the fistula, the left ventricular ejection fraction (LVEF) decreased from 51% preoperatively to 40% before discharge. It is possible that a long-term existence of a large fistula and left-to-right shunt led to chronic myocardial hypoperfusion and heart dysfunction, resulting in an impaired bearing capacity of surgical trauma and a longer recovery time. After discharge, the patient recovered well without any symptoms, and LVEF increased to 52% at 7 months post-operation.

### Histological studies

A total of 4% paraformaldehyde-fixed and paraffin-embedded right coronary artery aneurysms from the patient were cut into 4 μm sections. Standard hematoxylin and eosin (HE) and Alizarin red staining were used for histological evaluation.

### Whole exome sequencing, data analysis, and processing

The patient and his mother agreed to participate in the study; however, his father refused. Peripheral venous blood samples were collected from the patient and his mother after obtaining their written informed consent. Genomic DNA was isolated using the QIAamp DNA Blood Mini kit (Qiagen, Duesseldorf, Germany) according to the manufacturer’s instructions. His father refused to participate in the genetic testing, thus we did not obtain a DNA sample. Exome sequencing was performed by a commercial provider (Sinotech Genomics Co. Ltd., Shenzhen, China). Standard instructions from the manufacturer were used for capture with the SureSelect Human All Exon V6 kit (Agilent Technologies, Santa Clara, CA, USA), and 100 bp paired-end sequencing reads on the Illumina HiSeq 2500 platform, followed by bioinformatics processing and variant annotation as previously described (14). Raw data were aligned to the human reference genome (hg19). The mapping ratio was above 99% and the mean depth of coverage was ≥100X. Among all the bases in the captured target area, the percentage of coverage ≥10X was 95%. All identified variants were considered *a priori* variants of uncertain significance (VUS). Variants predicted to result in prematurely truncated proteins (non-sense, frameshift mutations, and affecting initiation codon) and canonical splice site variants (±2 bp) were given the highest priority.

*In silico* tools, including MutationTaster, SIFT, PolyPhen2, MutationAssessor, FATHMM, CADD, and GERP++ software, were used to evaluate possible pathogenic effects of the identified variants.

### Sanger sequencing analysis

Sanger sequencing was performed to validate candidate variants. Primer premier5 software was used to design the PCR primers. The PCR products were sequenced using an ABI 3730XL sequencer, and the results were compared to the reference *NEXN* sequence (NM_144573) and *ABCC6* sequence (NM_001171) using the GenBank BLAST program.^1^

## Results

The RCAF manifestations and cardiac functions of the patients are shown in Figure 1 and Table 1. There was no significant wall thickening or abnormality in the excised coronary artery aneurysm detected by HE and Alizarin red staining (Figures 1F,G). The diameter of the heart (LVDD) was significantly reduced after surgery, and heart function returned to preoperative levels by the 7-month follow-up (Table 1).

**TABLE 1:** Cardiac phenotypes of the patient carrying NEXN-G100X.

	LVDD (mm)	LVEF (%)	LVFS (%)	AI	Fistula (mm)	PDA (mm)
Pre-operation	57.2	51	27	Mild	7.6	1
Before discharge	48.3	40	19	Mild	None	None
Follow-up*	49	52	26	None	None	None

LVDD, left ventricular diastolic diameter; LVEF, left ventricular ejection fraction; LVFS, left ventricular shortening fraction; AI, aortic insufficiency; PDA, patent ductus arteriosus.

*7 months post-operation.

**FIGURE 2:**
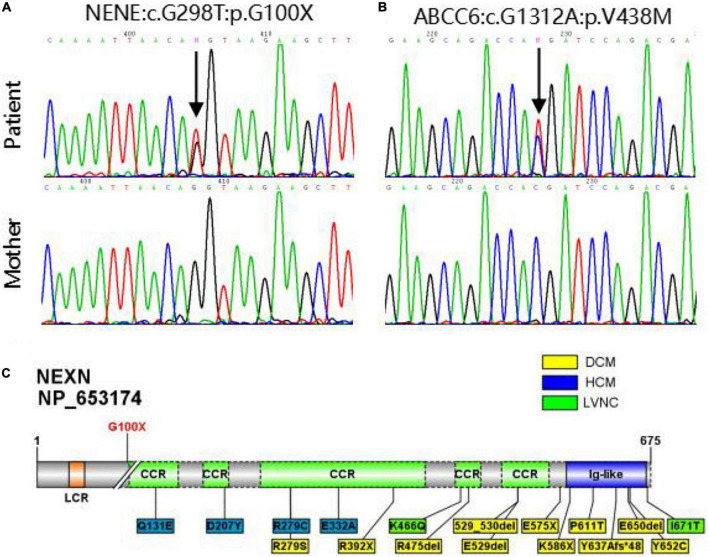
**(A,B)** Sanger sequencing validation of NEXN variant and ABCC6 variant in the patient and his mother. **(C)** Diagram of NEXN protein with the location of variant identified in this study (upper). All 17 variants in NEXN (NM_144573, NP_653174) previously reported cardiomyopathy patients are included in the schematic (underneath). The yellow and blue boxes indicate that the patient has dilated cardiomyopathy (DCM) and hypertrophic cardiomyopathy (HCM), respectively, and the green one indicates that the patient has left ventricular non-compaction (LVNC).

Using whole exome sequencing (WES), we observed a heterozygous truncated variant (NM_144573: c.G298T; p.G100X) in the *NEXN* gene and a missense variant in the *ABCC6* gene carried by the patient but not his mother (Table 2 and Figure 2). The NEXN-G100X variant has not been reported previously. The ABCC6-V438M (NM_001171: c.G1312A; p.V438M, rs542502733) variant was found in the NCBI, SNP, ExAC, and gnomAD databases, with a MAF of 0.0041 in East Asians.

**TABLE 2:** Mutations carried out by the patient identified by the WES analysis.

Gene	Transcription	Nucleotide change	Amino acid change	Zygosity	SNP	ExACEAS	gnomADEAS	SIFT	Polyphen2	MutationTaster	InterVar
NEXN	NM_144573	c.G298T	p.G100X	Het	/	/	/	/	/	D (1)	P
ABCC6	NM_001171	c.G1312A	p.V438M	Het	rs542502733	0.0041	0.0041	D (0.043)	D (0.978)	D (1)	US

D, deleterious; T, tolerated; B, benign; P, pathogenic; US, uncertain significance; Het, heterozygote.

## Discussion

In this study, we performed a successful surgical repair in a 6-year-old patient with a right coronary artery to right ventricle fistula (CAF), giant CAA, and PDA. Genetic evaluation and testing revealed that the patient harbored a novel NEXN-truncated variant, NEXN-G100X. CAFs were first described by German anatomist Krause in 1865 (4). The pathophysiological changes caused by CAF depend mainly on resistance (which is determined by the length, size, and tortuosity of the fistula) and on the site of drainage (10, 15). When the drainage site is located in the left atrium or pulmonary vein, an effective left-to-left shunt occurs and creates a volume overload in the left heart only (10, 15). When the fistula flows down to the left ventricle, it produces blood run-off from the aorta, simulating aortic valve regurgitation and leading to a volume overload of the left chambers. When the fistula flows down to the right vessels/chambers, a left-to-right shunt develops with volume overloading of both ventricles, which exists in over 90% of cases (15). In these conditions, especially in large CAFs, a diastolic “coronary steal” may exist, drawing blood away from the normal coronary circulation and myocardial microcirculation, leading to symptoms and signs of myocardial ischemia. In addition, persistent high flow in the coronary arteries may cause massive dilatation and aneurysm formation (15). In our case, blood flow from the right coronary artery to the right ventricle occurred throughout the cardiac cycle. The right coronary artery was dilated and aneurysmal. Although the patient did not show “coronary steal,” it was necessary to close the fistula due to its large size and secondary aneurysm.

Frequently isolated (80%) CAFs can also be associated with other cardiac malformations (20%), mostly atrial or ventricular septal defects, tetralogy of Fallot, and PDA (15). In addition, CAFs might be secondary to pulmonary atresia, intact ventricular septum (PA-IVS), and isolated valvular pulmonary stenosis (PS) (16). The coexistence of PDA and coronary artery fistula is very rare, and only a few cases have been reported previously (17–19). The first successful surgical closure of a coronary fistula was performed in 1947 by Bjork and Crafoord in a patient with a preoperative diagnosis of patent ductus arteriosus (1). In our patient, the PDA was tiny, with a 1.0 mm shunt, and was ligated during the surgery.

Coronary development involves an intricately timed series of events, including vasculogenesis, angiogenesis, and/or arteriogenesis (6). During the early embryonic stage, the heart consists of a thin myocardial muscle layer that is easily oxygenated by the diffusion of blood in the cardiac lumen. Later, as the heart size increases, the ventricular wall becomes thicker, and oxygen supply through passive diffusion is insufficient to supply the thickening myocardial wall. The coronary vascular plexus begins to emerge on the ventricles and migrates over the surface of the heart and into the myocardium (vasculogenesis). The plexus vessels then attach to the aorta to initiate blood flow, triggering arterial remodeling that ultimately leads to mature arteries (angiogenesis) (20). Arteriogenesis, following coronary arterial obstruction, involves the creation of mature vessels from pre-existing interconnecting arterioles (6).

Several key events occur during coronary artery development, the alteration of which leads to coronary artery anomalies. First, the coronary artery stems are connected to the cardiac arterial pole. Second, the differentiation of coronary cell progenitors occurs. Third, there is an interaction between the coronary vessels and the myocardium. Disruption of these processes could result in (i) anomalies of origin and course (such as the left coronary artery connecting to the pulmonary artery), (ii) anomalies of intrinsic coronary artery anatomy (such as coronary artery ostial stenosis or atresia), and (iii) anomalies of termination (such as myocardial bridge and CAF) (10). Several molecules and cellular signaling pathways are linked to proper coronary artery development, including the vascular endothelial growth factor (VEGF) family, ELA-APJ signaling axis, CXCL12/CXCR4 signaling axis, connexin43, Ino80, Tbx1, and Pofut1 (21–28). These studies provide evidence for genetic screening that may detect the genetic basis of coronary artery anomalies and provide insight for individuals at risk of coronary anomalies.

The genetic evaluation was performed on a 6-year-old CAF patient and his mother. WES and Sanger sequencing revealed a novel truncated G100X variant in NEXN. Other potential causes of the coronary anomalies were excluded. NEXN (Nexilin) is an F-actin binding protein present at the cell-matrix junction and functions in cell adhesion and migration. Previous studies have shown that NEXN stabilizes the Z-disk for force generation in adult cardiomyocytes. Several loss-of-function mutations in *NEXN* have been identified in both dilated cardiomyopathy and hypertrophic cardiomyopathy. Mutant NEXN exhibited impaired actin-binding ability or destabilization of the Z-disk in patients (29, 30). Expression of NEXN is detectable at an early stage of cardiac development, and cardiac-selective expression of NEXN negatively regulates cardiac differentiation. Additionally, it is suggested to lead to ASDs by inhibiting the cardiac key transcription factor GATA4 in mice and humans (13). Heterozygous variants of NEXN have previously been described in dilated cardiomyopathy (DCM) and hypertrophic cardiomyopathy (HCM) patients at a mean age of 50 years (31–33). Our patient, who carried the NEXN-G100X truncated variant, was 6 years old and had cardiomegaly with a slightly reduced cardiac function (EF = 51%). Regular follow-up is necessary to monitor cardiac phenotypes. However, NEXN has not been reported to be associated with coronary artery development. As mentioned above, an anomalous myocardial-coronary artery interaction leads to a fistula. NEXN is involved in cardiac differentiation and ventricular myocardial maturation. The NEXN-G100X mutants may affect cardiac myocardial development and myocardial-coronary interactions. Additionally, NEXN is involved in the VEGF-A-VEGFR2 signaling pathway. Myocardial VEGF-A is important for the development of the intramyocardial network. VEGFR2 is highly expressed at the site where the primitive capillary plexus invades the aorta to form the major CA. Previous studies on VEGF-A and VEGFR2 null hearts indicate a hypoxic-dependent VEGF-A concentration gradient in the myocardium of the early heart, which promotes endocardial sprouting into the ventricular walls, contributing to CA development (6, 34, 35). The VEGF signaling pathway regulates coronary smooth muscle cell differentiation to coordinate inner and outer coronary vessel wall morphogenesis (36). NEXN is highly expressed in smooth muscle cells (SMCs), including coronary artery SMCs. A previous study has shown that NEXN is a dense body/dense band-associated protein in SMCs that promotes actin polymerization and cell migration and amplifies SMC differentiation (37). The development of a mature, definitive coronary tree is dependent on medial coronary SMC differentiation and endothelial-SMC molecular crosstalk. Alterations in this process are likely to affect the morphology and viability of early coronary artery blood vessels (10). Based on the reports above and our WES results, we speculated that NEXN may be associated with coronary development. Further studies should be carried out to examine the coronary artery phenotypes in *NEXN* knockout mice and to provide robust evidence.

ATP binding cassette subfamily C member 6 (ABCC6) belongs to the multidrug resistance-associated protein (MRP) subfamily. *ABCC6* encodes a 1503 amino acid transmembrane protein. Mutations in ABCC6 are a major pathogenic factor in the development of pseudoxanthoma elasticum (PXE), a heritable connective tissue disorder characterized by the calcification of elastic fibers in the skin, arteries, and retina (38). Although the patient carried the heterozygous ABCC6-V438M variant, he did not show calcification of the skin or arteries. The patient needs to be followed up, and the effect of the mutation remains to be elucidated.

## Conclusion

In conclusion, we report a novel NEXN-truncated variant NEXN-G100X in a patient with right coronary artery to right ventricle fistula (CAF), giant CAA, and PDA. There are rare reports of genetic abnormalities of congenital coronary artery-to-ventricle fistulas combined with giant coronary artery aneurysms. Our findings may be useful in understanding the mechanisms underlying coronary artery fistula and giant coronary artery aneurysms. Further genomic and functional studies are needed to better delineate the underlying mechanisms of CAF and its correlation with NEXN.

## Data availability statement

The original contributions presented in this study are publicly available. This data can be found here: https://db.cngb.org/cnsa/, accession number: CNP0003648.

## Ethics statement

The studies involving human participants were reviewed and approved by Xinhua Hospital Ethics Committees. Written informed consent to participate in this study was provided by the participants’ legal guardian/next of kin.

## Author contributions

YL, SC, and KB contributed to the study’s conception and design. YP, JH, YW, and WL organized the clinical database. JY and YX performed the histological and exome sequencing analyses. YP and JY wrote the first draft of this manuscript. All authors contributed to the manuscript revision and read and approved the submitted version.

## Footnotes

1
http://blast.ncbi.nlm.nih.gov/Blast.cgi
